# CRISPR/Cas9 Mediated Knockout of the *OsbHLH024* Transcription Factor Improves Salt Stress Resistance in Rice (*Oryza sativa* L.)

**DOI:** 10.3390/plants11091184

**Published:** 2022-04-27

**Authors:** Mohammad Shah Alam, Jiarui Kong, Ruofu Tao, Temoor Ahmed, Md. Alamin, Saqer S. Alotaibi, Nader R. Abdelsalam, Jian-Hong Xu

**Affiliations:** 1 Institute of Crop Science, Zhejiang Key Laboratory of Crop Germplasm, Zhejiang University, Hangzhou 310058, China; salam9488@yahoo.com (M.S.A.); kjr1234@zju.edu.cn (J.K.); m18805817913@163.com (R.T.); alamin@zju.edu.cn (M.A.); 2 Institute of Biotechnology, Zhejiang University, Hangzhou 310058, China; temoorahmed@zju.edu.cn; 3 Department of Biotechnology, College of Science, Taif University, P.O. Box 11099, Taif 21944, Saudi Arabia; saqer@tu.edu.sa; 4 Agricultural Botany Department, Faculty of Agriculture (Saba Basha), Alexandria University, Alexandria 21531, Egypt; nader.wheat@alexu.edu.eg; 5 Shandong (Linyi) Institute of Modern Agriculture, Zhejiang University, Linyi 276000, China

**Keywords:** antioxidants, CRISPR/Cas9, rice, *OsbHLH024*, salt stress, ROS

## Abstract

Salinity stress is one of the most prominent abiotic stresses that negatively affect crop production. Transcription factors (TFs) are involved in the absorption, transport, or compartmentation of sodium (Na^+^) or potassium (K^+^) to resist salt stress. The basic helix–loop–helix (bHLH) is a TF gene family critical for plant growth and stress responses, including salinity. Herein, we used the CRISPR/Cas9 strategy to generate the gene editing mutant to investigate the role of *OsbHLH024* in rice under salt stress. The A nucleotide base deletion was identified in the *osbhlh024* mutant (A91). Exposure of the A91 under salt stress resulted in a significant increase in the shoot weight, the total chlorophyll content, and the chlorophyll fluorescence. Moreover, high antioxidant activities coincided with less reactive oxygen species (ROS) and stabilized levels of MDA in the A91. This better control of oxidative stress was accompanied by fewer Na^+^ but more K^+^, and a balanced level of Ca^2+^, Zn^2+^, and Mg^2+^ in the shoot and root of the A91, allowing it to withstand salt stress. Furthermore, the A91 also presented a significantly up-regulated expression of the ion transporter genes (*OsHKT1;3*, *OsHAK7*, and *OsSOS1*) in the shoot when exposed to salt stress. These findings imply that the *OsbHLH024* might play the role of a negative regulator of salt stress, which will help to understand better the molecular basis of rice production improvement under salt stress.

## 1. Introduction

Salt stress is significant abiotic stress that affects plant growth and development and renders natural production unfeasible [1]. Although rice (*Oryza sativa* L.) is an essential crop for feeding over half of the earth’s population, its production is limited by salt stress. Various parameters are disrupted under salinity stress at the physiological level, for instance, the stomatal closure and chlorophyll content inhibition resulted in the reduction of the photosynthesis [2]. Moreover, oxidative damage, nutrient imbalances, toxic metabolite formation, and cellular damage are often associated with plant growth inhibition and the reduction of crop production [3,4]. Such physiological parameters require particular consideration for uncovering the tolerance mechanism of salt stress in rice.

Reactive oxygen species (ROS) and ionic imbalance play a critical role in the response to salt stress [5]. ROS, including hydrogen peroxide (H_2_O_2_), superoxide radicals (O_2_^−^), hydroxyl radicals (OH^.^), and singlet oxygen (^1^O_2_), are generated in different cellular compartments of plants under salt stress [6]. Under aerobic conditions, ROS are produced from mitochondrial and chloroplast electron transport chains [7] and controlled through different antioxidant defense systems [8,9]. Salt stress can quickly initiate and increase ROS production, responsible for oxidative damage [10], lipid peroxidation (malondialdehyde, MDA), membrane injury [11], and eventually cell death [12]. Antioxidants are essential for scavenging ROS, and ROS mitigation occurs in plant cells [13]. Superoxide dismutase (SOD), a metalloenzyme that dismutases ROS into H_2_O_2_ and O_2_, is a frontline defense against ROS and peroxidases (POD), converting H_2_O_2_ to H_2_O [14]. The salt stress toxicity is mainly due to Na^+^ and Cl^−^ toxicity, which can induce remarkable disturbance of important nutrients, including N, P, K^+^, Ca^2+^, S, Mn^2+^, Mg^2+^, and Zn^2+^ [3,15]. Therefore, the salt stress can restrict the Ca^2+^ absorption, reduce cytosolic Ca^2+^ levels, affect membrane stability, and influence the osmotic equilibrium and intracellular signaling [16]. Furthermore, Zn^2+^ can also improve the salt stress tolerance by blocking Na^+^ and/or Cl^−^ absorption or translocation [17]. However, the Zn^2+^ assimilation in plants is also inhibited by greater salt concentrations [18]. Development of the varieties with enhanced capacity for ROS scavenging and ionic homeostasis [19] could pave the way to improving rice tolerance towards salt stress. 

Some genes are involved in salt stress regulation, including genes associated with blocking Na^+^ entrance, translocation, and/or transport out of cells [4], and genes codifying the signaling of proteins responsible for ROS scavenging to regulate the proper nutrients balance [20]. The high-affinity potassium transporters (HKTs), KT/HAK/KUP family, Salt Overly Sensitive (SOS), as well as late embryogenesis abundant (LEA) have been reported to play a vital role in the salt stress response [21,22,23,24]. Among them, HKTs regulate the balance of Na^+^ and K^+^ [25], which can be divided into HKT1, a low-affinity Na^+^ transporter, and HKT2, a permeable channel for Na^+^ and K^+^ [26]. Under salt stress, *OsHKT1* excludes Na^+^ from root xylem sap to prevent it reaching the shoots or its translocation from shoots to roots to promote efflux [27]. In rice, *OsHKT1;1* loads Na^+^ into parenchyma cells that might be transferred to the phloem after being transported to the leaves [28]. Moreover, *OsHKT1;1* recovers Na^+^ from the leaf blade and is mainly expressed in roots, vascular tissues, and leaves [28], but the exact role regarding salinity tolerance is unknown [29]. The *OsHKT1;3* transporter is a highly selective Na^+^ transporter found in the vascular tissue of roots and leaves [30], and it is significantly expressed when exposed to salt [31]. *OsHKT1;5* (rice QTL, SKC1) controls Na^+^ concentration in the xylem of leaves and is expressed in roots and shoots [32]. On the other hand, the *OsHKT2* facilitates the uptake of Na^+^ or K^+^ into starved tissues in rice [33]. The KT/HAK/KUP family of K^+^ transporters are also essential genes involved in the salt stress response, among which *OsHAK7* is a salinity-resistant gene found in the shoots and roots of plants [34]. Furthermore, *SOS1* functions in the SOS signaling system in plasma membranes, and its mutation, *sos1*, imparts salt sensitivity by allowing more Na^+^ and less K^+^ to enter the cell [35]. The late embryogenesis abundant group 3 protein in rice (*OsLEA3)* presents a protective function under extreme stress circumstances, including salt stress [21]. Thus, investigation of these genes relevant to the plant response to salt stress will help to elucidate the underlying mechanisms.

The defense mechanism could be controlled by regulatory elements, including transcription factors (TFs) [36]. TFs bind to *cis*-acting domains in promoters of target genes to regulate gene expression positively or negatively [37]. The regulation of TFs is one of the major mechanisms by which plants ensure growth, development, and protection from numerous environmental stresses [38]. The plant basic-helix–loop–helix (bHLH) is one of the largest TFs, compromising 25 subfamilies functioning against abiotic stress, including salt stress [39,40]. *AtbHLH092*, *AtbHLH112* in *Arabidopsis* [41,42], *PvbHLH*-*54*, *PvbHLH*-*148* in common bean [43], *VvbHLH1* in grape [44], *BvbHLH93* in sugar beet [45], *SbbHLH85* in sweet sorghum [46], and *NtbHLH123* in tobacco [47] have been reported to play a role in the salt stress response. Only three bHLH genes, *OsbHLH035*, *OsbHLH062*, and *OsbHLH068*, have been associated with the rice response towards salt stress [42,48,49], which belong to subfamilies V, M, and F, respectively. However, the function of the bHLH subfamily U has not been explored yet. The rice bHLH subfamily U includes seven genes [39], and the representative *OsbHLH024* (*LOC_Os01g39330*) gene was selected to address the function of the bHLH subfamily U response to salt stress in rice. The *OsbHLH024* differentially expressed between high temperature and low temperature in thermosensitive genic male-sterile material Zheda13S [50]. In this study, to investigate the function of the *OsbHLH024* gene in response to salt stress, a loss-of-function mutant was generated using the CRISPR/Cas9 approach. Interestingly, the *osbhlh024* mutant (A91) exhibited a high salinity tolerance response, and accumulated less ROS, MDA, and Na^+^ but a higher K^+^ with a balanced nutritional level in the shoots and roots. Moreover, the expression of ion transporter genes of *OsHKT1;3*, *OsHAK7*, and *OsSOS1* was enhanced in the A91 under salt stress. These findings suggest that the *OsbHLH024* gene is a negative regulator, and its knockout enhances the salt stress tolerance in rice.

## 2. Results

### 2.1. Expression Level of OsbHLH024

**Figure 1:**
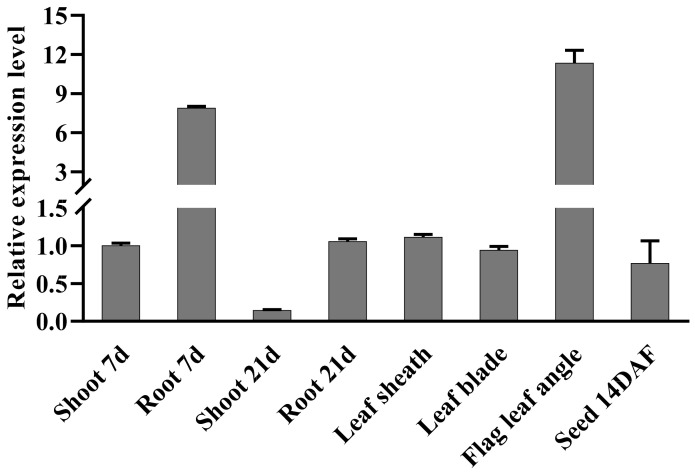
The expression level of *OsbHLH024* in eight different tissues. *OsUBQ5* was used as the control; three biological replicates were applied, and the values were expressed as means ± SE.

Eight different tissues of rice wild type (WT) Nipponbare were sampled to investigate the expression level of *OsbHLH024* gene by qRT-PCR. The results showed that *OsbHLH024* was highly expressed in the flag leaves angle and roots at 7 days. While other tissues had a low expression, shoots investigated at 21 days showed very low expression levels (Figure 1).

### 2.2. Generation of A91 via CRISPR/Cas9 Method

**Figure 2:**
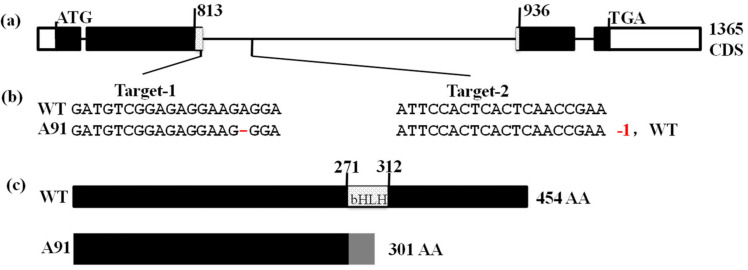
CRISPR/Cas9-induced mutation in the *OsbHLH024* (*LOC_Os01g39330*) gene. (**a**) Schematic diagram of gene structure and two CRISPR/Cas9 target locations, with UTRs, exons, and introns and bHLH domain shown by blank rectangles, black rectangles, black lines, and dotted rectangles, respectively. The 20-nt target sequences are shown at the bottom of the gene structure; (**b**) DNA sequencing and alignments with WT identify the editing genotypes; the deletion is indicated by the red dash; (**c**) the expected protein structure of WT and A91. The dotted and grey showed the bHLH domain in WT and frameshift or premature stop in A91.

For characterizing the function of the *OsbHLH024* gene in rice, we created the A91 using the CRISPR/Cas9 genome editing method. Two single guide RNA (sgRNAs) in two targets with sequence-specific sites of *OsbHLH024* were designed to induce mutagenesis. The target-1 and target-2 were separated by 312 bp and driven by OsU6a and OsU6b promoters, respectively (Figure 2a and Figure S1). They were ligated and inserted into a CRISPR/Cas9 binary vector through two sgRNA expression cassettes [51]. Using *Agrobacterium* induced transformation, the final vector was transformed into the calli of WT Nipponbare. The generated transgenic plants were analyzed using PCR with site-specific (target) primers and Sanger sequencing to confirm the gene-editing type. Only one heterozygous mutant was obtained, and the homozygous mutant (A91) with one base (A) deletion in Target-1 in T_1_ generation was obtained (Figure 2b and Figure S2), which resulted in a frameshift to accumulate the stop codon (premature) with 301 of the original 455 amino acids (Figure 2c).

### 2.3. Phenotypic Characterization of the A91 and WT Grew in Normal Conditions

**Figure 3:**
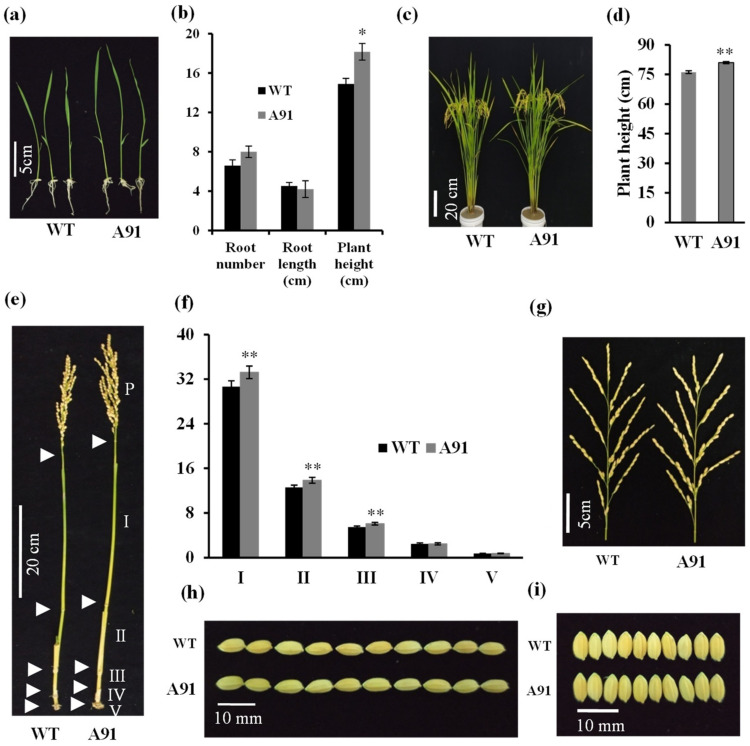
The phenotypic comparison of the A91 and WT. (**a**) The height of seedlings at one-week-old growing in ½ MS media; (**b**) the comparison of the root number, the root length and the shoot length in WT and A91 (n = 9 biological replicates); (**c**) the morphology of plants at the reproductive stage; (**d**) the measurement of plant height (n = 30 biological replicates); (**e**) the phenotype of tiller; (**f**) the measurement of internode length (n = 30 biological replicates); (**g**) the structure of panicle; (**h**) 10 seeds length; (**i**) 10 seeds width, values are expressed as means ± SE; * and ** denote significant *t*-test results at *p* < 0.05 and *p* < 0.01, respectively.

At the one-week seedling stage, the plant height was significantly different between WT (14.90 cm) and the A91 (18.16 cm) (Figure 3a,b). The difference in the root number and root length was insignificant between WT and A91 (Figure 3b). At the reproductive stage, the plant height was raised by 6.13% in the A91 compared to the WT (Figure 3c,d). Both the WT and the A91 presented five internodes, and the first, second, and third internodes of the A91 were notably longer than that of the WT, while the fourth and fifth internodes were unchanged (Figure 3e,f). The tiller biomass was increased by 14.18% in the A91 compared to the WT (Table S2). On the other hand, the tiller number, panicle size, grain shape, and leaf size remained statistically stable in the A91 and WT (Figure 3g–i and Table S2). These observations indicated that the A91 exhibits fewer effects on the rice phenotypes and could be characterized clearly by the plant height and tiller biomass at the seedling and reproductive stages under normal conditions.

### 2.4. The A91 Confers Salt Tolerance

**Figure 4:**
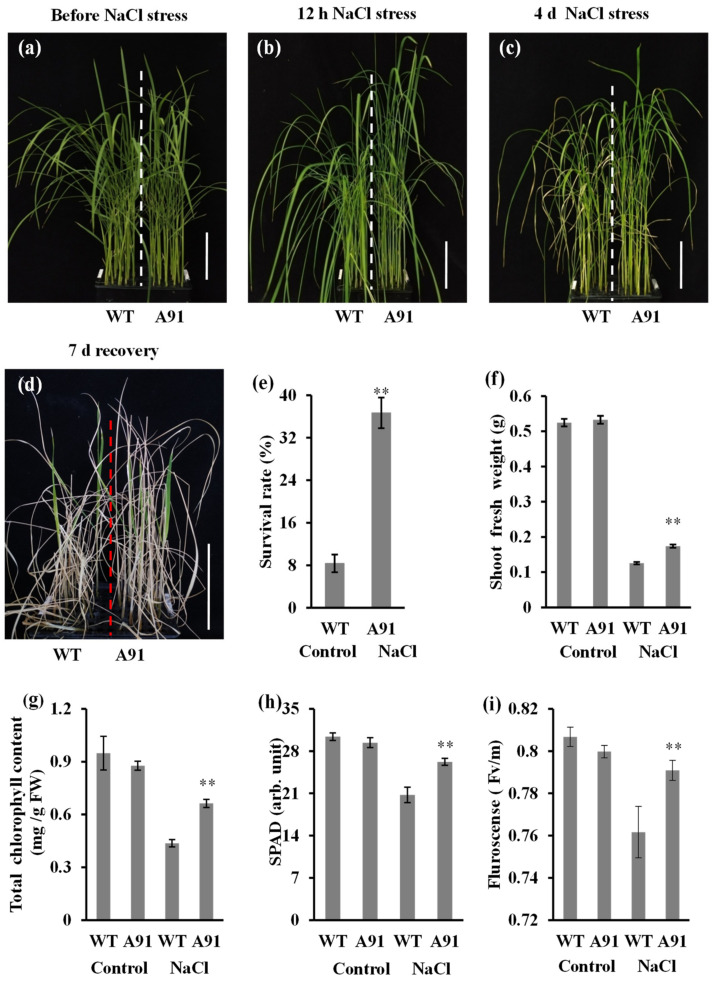
The growth characteristics of the A91 and WT exposed to salt stress treatments (0 and 150 mM NaCl). (**a**) The growth of 21-day-old seedlings before salt stress; (**b**) the growth of 21-day-old seedlings after 12 h stress; (**c**) the growth of 21-day-old seedlings after 4 days of stress; (**d**) 7 days recovery after 7 days of salt stress; (**e**) the survival rate after 7 days of recovery; (**f**) the fresh shoot weight after 7 days of stress; (**g**) total chlorophyll content; (**h**) SPAD value; (**i**) fluorescence; n = 3 biological replicates (**e**,**f**, each replicate represented 20 seedlings); 3 biological replicates (**g**), 9 biological replicates (**h**,**i**); data represented as means ± SE; ** indicates significant *t*-test results at *p* < 0.01; scale bar 10 cm.

The 21-day-old seedlings of the A91 and WT were exposed to 150 mM NaCl (Figure 4), and most of the WT seedlings collapsed after 12 h of salt treatment (Figure 4b). The prolongation of salt exposure resulted in visually wilted, rolled, dried, stunted, and burnt leaves after 4 days (Figure 4c). Although the stress symptoms appeared after 6 days and became clear after 7 days of salt stress exposure (Figure S3), the A91 exhibited better growth performance than the WT (Figure 4a–c). Moreover, the A91 presented a greater survival rate (7.33/20, 36.67%) than the WT (1.66/20, 8.33%) after 7 days of recovery (Figure 4d,e). While salt stress markedly decreased the growth performance of both the WT and the A91, the fresh shoot weight of the mutant was considerably higher than that of the WT after 7 days of salt exposure (Figure 4f). 

The total chlorophyll content (SPAD value) and chlorophyll fluorescence were significantly greater in A91 than in WT after 4 days of salt stress exposure (Figure 4g–i). These results suggested that the knockout of the *OsbHLH024* gene confers the salt tolerance in rice by improving the physiological condition of leaves, delaying the appearance of stress symptoms, and consequently promoting better shoot performance and survival rate.

### 2.5. OsbHLH024 Involves in the Regulation of Oxidative Stress Homeostasis in Salt Stress 

**Figure 5:**
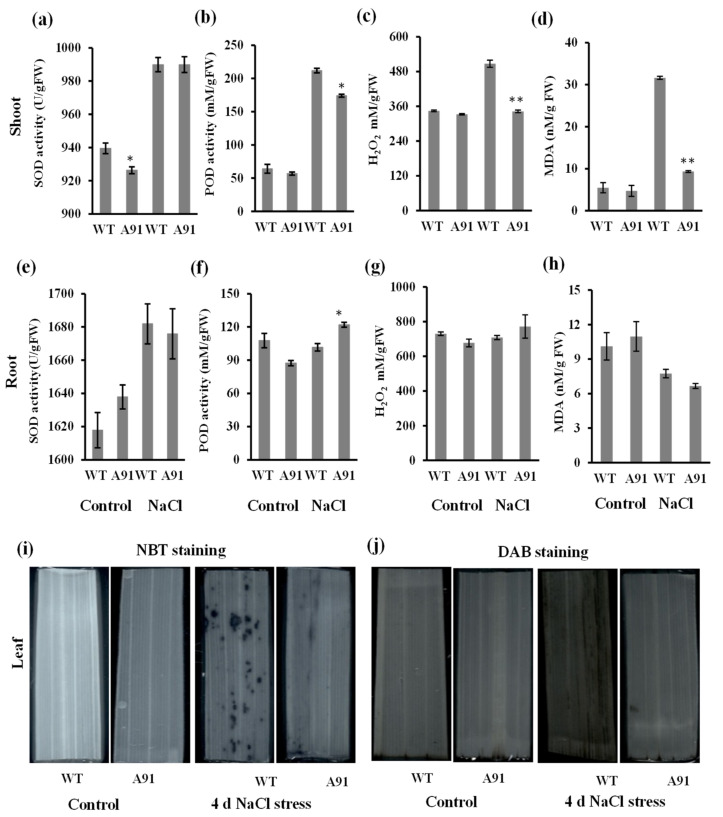
The oxidative stress in the shoot and root of WT and A91 seedlings under salt stress. (**a**–**d**) activities of SOD, POD, H_2_O_2,_ and MDA in shoot; (**e**–**h**) activities of SOD, POD, H_2_O_2,_ and MDA in the root; (**i**) NBT staining (indicates O_2_^•−^); (**j**) DAB staining (indicates H_2_O_2_); n = 3 biological replicates, data represent means ± SE; * and ** indicate significant *t*-test results at *p* < 0.05 and *p* < 0.01, respectively.

Because salt stress is often accompanied by oxidative stress [3], the antioxidant enzyme (SOD and POD) activities and the levels of H_2_O_2_, MDA, and superoxide radicals (O_2_^•−^) were further investigated in shoots and roots of the A91 and WT. Although salt stress significantly increased the SOD activity in both the A91 and WT, the level of SOD activity was not significantly different between the A91 and WT (Figure 5a,e). The SOD activity regulates the production of H_2_O_2_, the H_2_O_2_ in the shoot of WT was greater than in the A91 under salt stress (Figure 5c). In addition, the salt stress coincided with a significant rise in the POD activity but at a lower level in the shoots of A91 than WT (Figure 5b). Compared to their respective controls, POD activity increased more in the shoots of WT than the A91 as a response to the H_2_O_2_ level (Figure 5b,c). Interestingly, no significant difference in the MDA level was recorded in the shoots of WT and A91 under control, while it was 3.8-fold higher in WT than in A91 under salt stress (Figure 5d). The MDA level in roots did not differ significantly before and after the salt exposure (Figure 5h). These physiological observations suggested the significance of salt stress in the shoots of WT compared to the A91. Moreover, the POD activity was more induced in the roots of the A91 than the WT under salt stress and indicated that the response of POD depended on the level of H_2_O_2_ in the roots (Figure 5f,g).

Furthermore, in situ histochemical DAB and NBT staining were employed to measure H_2_O_2_ and O2^•−^, respectively. The highly dark-brown color and more staining spots were displayed in the WT leaf than that of A91 after 4 days of salt stress (Figure 5i,j), supporting that the WT accumulated more H_2_O_2_ and O_2_^•−^ than the mutant. These results revealed the significance of oxidative stress due to salt stress in the WT compared to the A91.

### 2.6. OsbHLH024 Induces Ion Homeostasis under Salt Stress 

**Figure 6:**
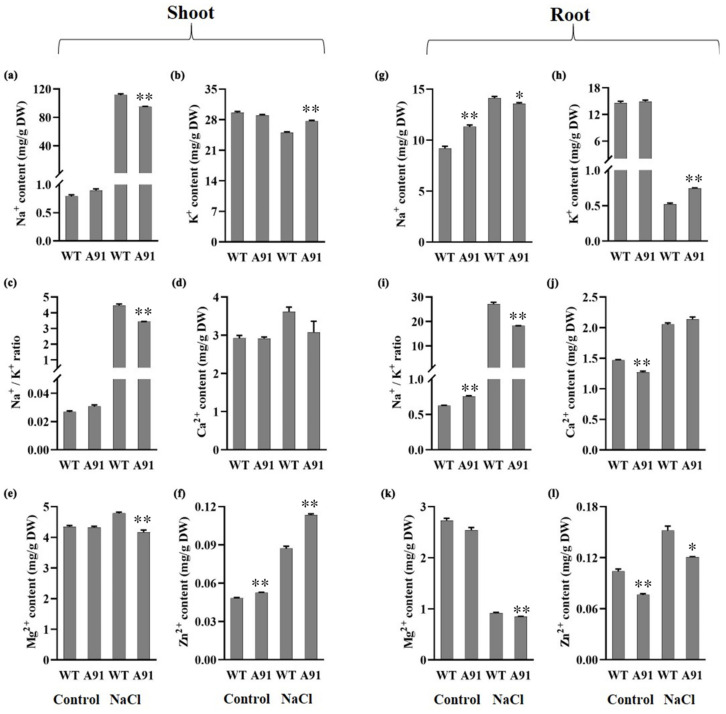
Measurement of nutrient elements in the shoots and roots of WT and A91 seedlings exposed to salt stress for 7 days. (**a**–**f**) Na^+^, K^+^, Na^+^/K^+^, Ca^2+^, Mg^2+^, and Zn^2+^ in shoots; (**g**–**l**) Na^+^, K^+^, Na^+^/K^+^, Ca^2+^, Mg^2+^, and Zn^2+^ in roots; n = 3 biological replicates; data represent means ± SE; * and ** indicate significant *t*-test results at *p* < 0.05 and *p* < 0.01, respectively.

The levels of Na^+^, K^+^, Ca^2+^, Mg^2+^, and Zn^2+^ in the shoots and roots of WT and A91 were studied before and after salt stress. Before salt stress, only the Zn^2+^ level in the shoots was significantly higher in the A91 than that of WT (Figure 6a–f). After 7 days of salt exposure, a low level of Na^+^ and Mg^2+^ was found in the shoots of A91 seedlings compared to the WT (Figure 6a,e). By contrast, the content of K^+^ and Zn^2+^ levels were greater in the shoots of the A91 than in the WT under salt stress (Figure 6b,f). Interestingly, a lower ratio of Na^+^/K^+^ was observed in the shoots of the A91 than in WT (Figure 6c). Ca^2+^ was not significant before and after salt stress, although there was an increasing trend observed after stress in both WT and A91 (Figure 6d). These results indicated a lower Na^+^ translocation in the shoots of the A91 compared to WT.

The salt stress resulted in a higher Na^+^ content in the roots of WT than in the A91, but compared to WT, the A91 showed higher Na^+^ content in control (Figure 6g). Although the content of K^+^ in the roots was significantly decreased by salt exposure, the level of K^+^ in the A91 was higher than in WT (Figure 6h). Under salt stress, the ratio of Na ^+^ /K^+^ in the A91 was lower than in the WT, suggesting a superior uptake of Na^+^ in the WT than in the A91 (Figure 6i). Moreover, salt stress increased the level of Ca^2+^ and Zn^2+^ in the roots of both WT and A91 (Figure 6j,l). By contrast, the uptake of Mg^2+^ in both WT and A91 was repressed by the salt stress, which was significantly lower in A91 compared to WT (Figure 6k). These results showed that the A91 exhibited better nutritional homeostasis than WT under salt stress.

### 2.7. The Molecular Effect of OsbHLH024 Mutation in Ion Transporters and Cell Structure Controller Genes

**Figure 7:**
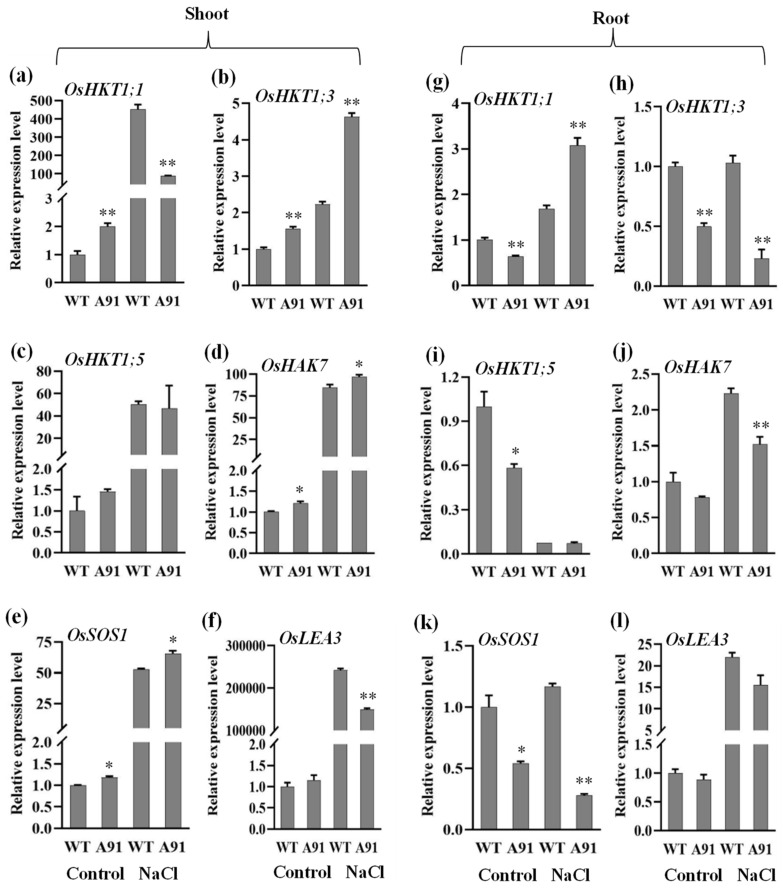
Expression of genes in WT and A91 under control and salt stress for 2 days. (**a**–**f**) *OsHKT1;1*, *OsHKT1;3*, *OsHKT1;5*, *OsHAK7*, *OsSOS1* and *OsLEA3* in shoots; (**g**–**l**) *OsHKT1;1*, *OsHKT1;3*, *OsHKT1;5*, *OsHAK7*, *OsSOS1* and *OsLEA3* in roots; *OsACTIN1* was used as the control; n = 3 biological replicates, data represent means ± SE; * and ** indicate significant *t*-test results at *p* < 0.05 and *p* < 0.01, respectively.

As the results of the phenotypic, physiological, antioxidant activities and ion homeostasis above showed that the A91 could be tolerant to salt stress, the molecular effects of the *OsbHLH024* mutation were investigated through the expression of five ion transporters (*OsHKT1;1*, *OsHKT1;3*, *OsHKT1;5*, *OsHAK7*, *OsSOS1*) and one cell structure controller gene (*OsLEA3*), in shoots and roots under salt stress (Figure 7). The salt stress remarkably increased the expression levels of all the six genes in the shoots of both WT and A91 (Figure 7a–f). Compared to the WT, the relative expression levels of *OsHKT1;3*, *OsHAK7*, and *OsSOS1* genes in the A91 were relatively higher before and after NaCl treatment (Figure 7b,d,e). On the other hand, *OsHKT1;1* was expressed highly in the A91 before salt stress but significantly decreased after stress (Figure 7a). *OsLEA3* was not significant before salt stress but significantly decreased after stress in the A91 (Figure 7f). The expression of *OsHKT1;5* was not changed significantly between WT and the A91 (Figure 7c). 

Opposite to shoots, the expression of *OsHKT1;3*, *OsHAK7*, and *OsSOS1* genes were decreased in roots of the A91 when compared to WT, and *OsHKT1;5* and *OsLEA3* genes had similar trends, but not significant (Figure 7). The *OsHKT1;1* gene had lower expression before stress but higher expression after salt stress in the A91compared to WT (Figure 7g). All six genes had higher expression in shoots compared to roots under salt stress. These results suggested that the mutation in the *OsbHLH024* gene positively affects the expression of ion transporter genes, especially in shoots, which are essential for maintaining nutrient balance.

## 3. Discussion

The world’s rice production, a major concern for the human diet, is hampered by excessive salinity [52]. In previous studies, the role of salinity was reported to weaken the PSII activity via promoting the compensation of the pigments in chlorophyll by the secretion of ions, which affects the electron transport chain and causes PSII inhibition [53]. Besides, the lower photosynthesis due to the salt stress is the result of either the stomata closure that leads to a drop in the intercellular CO_2_ additional pressure or factors regarding non-stomata that involve the depletion of chlorophyll, electron transport reaction in photosynthesis, as well as fixation of carbon [54]. The rice salinity tolerance was improved by high chlorophyll content and the overexpression of *OsEXPA7* and *STRK1* in plants [55,56]. By contrast, the low chlorophyll content of *STRK1*-RNAi transgenic plants and *oshkt1;1* mutant has been associated with salinity sensitivity [28,56]. TFs such as bHLH are essential genetic components with a potential contribution to regulate stress tolerance in plants [57]. In rice, there are several members of the bHLH TFs, among which the U subfamily has not been investigated yet, concerning its role in salt tolerance. In this study we generated the *OsbHLH024* gene editing mutant by CRISPR/Cas9 method. We introduced CRISPR/Cas9-*OsbHLH024* vector into rice calli many times, but achieved one mutation, and it may have some problems with the gene editing of *OsbHLH024*. Our findings have demonstrated that the knockout of the *OsbHLH024* confers salt tolerance in rice. The mutant *osbhlh024* (A91) seedlings presented some remarkable phenotypes, including the delayed stress symptoms, the higher shoot weight, an improved survival rate and chlorophyll content under salt stress (Figure 4).

The main challenge for plants exposed to salt stress is detoxifying MDA and ROS (O_2_^−^ and H_2_O_2_) mainly by the SOD and POD enzymes [9]. Our results clarified that antioxidant activity in the A91 contributed to better regulation of ROS and MDA to alleviate the oxidative stress under salt stress (Figure 5). In general, the ROS (H_2_O_2_) levels were higher in roots than shoots under salt stress because the root is the first organ to sense the environment in plant [58]. This is supported by the observation indicating the higher SOD levels in roots than shoots, especially under salt stress by at least 1.69-fold (Figure 5e). In the shoots, ROS (H_2_O_2_) and MDA were significantly lower in the *osbhlh024* mutant than WT, which might be due to the significance of SOD activity in the *OsbHLH024* loss-of-function mutant (Figure 5a). Moreover, since O_2_^−^ belongs to the short-lived ROS, it quickly generates other major ROS, such as hydrogen peroxide (H_2_O_2_), hydroxyl radicals (OH**^.^**), perhydroxyl radicals (OH_2_**^.^**), and singlet oxygen (^1^O_2_) [59]. Hydroxyl radicals (OH**^.^**) and singlet oxygen (^1^O_2_) are responsible for lipid peroxidation by attacking polyunsaturated fatty acids (PUFA) [60], suggesting SOD of WT might not be enough to catalyze those ROS, resulting in higher MDA levels in shoots (Figure 5d). Furthermore, less O_2_^−^ and H_2_O_2_ in leaves of the A91 mutant than in the WT indicated the severity of oxidative stress in WT compared to the A91 (Figure 5i,j). On the other hand, in shoots, the POD activity was significantly lower in the A91 than WT, suggesting that the level of POD activity was dependent on the level of H_2_O_2_ detected in the tissues responding to the salt stress. Interestingly, the greater level of POD activity in shoots of WT than in the A91 (Figure 5b) coincided with a significant MDA level, demonstrating that salt stress was more toxic in WT than in the A91. In roots, POD was significantly higher in the A91 than in the WT, suggesting that it might act as an ROS scavenger. These results indicated that the SOD and POD activity might contribute to the production and regulation of several ROS in the shoots and roots by which the A91 maintained ROS homeostasis under salt stress. 

The strategy to make rice plants tolerant to salinity depends on the ability to exclude Na^+^ uptake from the shoots and to keep a low ratio of Na^+^/K^+^ [61]. Previous studies have reported the effect of salt stress mediated by a high intercellular Na^+^ level, which can dislocate the Ca^2+^ out of membranes and cause the K^+^ efflux as well as leakage from cells [62]. On the other hand, the K^+^ loss can be mitigated by Ca^2+^ [63], suggesting the necessity of regulating the interaction of Na^+^, K^+^ and Ca^2+^ in plants exposed to salt stress. The activation of the SOS pathway can contribute directly or indirectly to salinity tolerance via the control of Ca^2+^, K^+^ and Na^+^ homeostasis in plants [64]. Several studies have shown that overexpression of *OsSTLK*, *SAPK1* and *OsSTAP1* increased K^+^, and decreased Na^+^ and Na^+^/K^+^ ratio [65,66,67]. The root is the primary source where salinity-induced genes can restrict Na^+^ entrance into the xylem through the roots [68], and Na^+^ exclusion by the roots is the most efficient strategy for enhancing plant tolerance [69]. Our data indicated that a higher expression of *OsHKT1;1* could mediate the salinity tolerance in the A91 through the unloading of Na^+^ from roots (Figure 7g). This is in agreement with the role of *AtHKT1:1* in regulating the Na^+^ accumulation by roots in *Arabidopsis*, [70] and Na^+^ content in rice [71]. Moreover, the downregulation of *OsLEA3* and *OsHKT1;1* in shoots (Figure 7a,f) and *OsHKT1;3*, *OsHAK7*, and *OsSOS1* in roots (Figure 7h,j,k) of the *osbhlh024* mutant might not be involved in adaption to the salt stress. These genes might be regulated by other genes in specific tissues, such as those belonging to *bHLH* family. Therefore, the mutation in *OsbHLH024* might also function in the regulation of Na^+^ transportation from leaves to roots. Previous research found that the Egyptian Yasmine is more salinity tolerant than Sakha 102, but that in leaf sheaths and leaves, the expression level of *OsHKT1;5* was suppressed and that the *OsSOS1* expression was reduced in leaves and roots, implying that these genes have dual roles [72]. In addition, *HKT1* has tissue specificity known as vascular bundle or pericycle [73], mutation of *HKT1* under *sos2* and *sos3* mutants represses hypersensitivity to salt, indicating Na ^+^ /K^+^ homeostasis of plant cells is the coordination function of *HKT1* and the SOS pathway [74].

However, an elevated amount of Na^+^ generally increased the Na^+^ /K^+^ ratio and reduced K^+^, resulting in sensitivity [75]. The loss-of-function *osbhlh024* mutant, A91 performed lower Na^+^, higher K^+^, lower Na^+^/K^+^ ratio accumulation in both shoots and roots (Figure 6a–c,g–i). Earlier studies investigated that *OsJRL* overexpression increased the expression of the *OsHKT1;3* gene that conferred salinity resistance [76], the salinity sensitive *osgty-2* mutant showed low expression of *OsHKT1;3* [77], and loss-of-function *osrr9* and *osrr10* mutants performed salinity resistance with high expression of *OsHKT1;3* [78]. Significantly reduced expression of *OsSOS1* in the *tsd2* mutant made the plant sensitive to salinity [75], while overexpression of *OsTRM13* increased the *OsSOS1* gene expression to positively regulate tolerance of salt stress [79]. Furthermore, overexpression of *SAPK1* revealed greater expression of *OsSOS1* that turned to better performance upon salinity [66]. *OsHAK7* mediates K^+^ transport under salt stress to ensure plant resistance [80]. The *OsHKT1;3*, *OsHAK7*, and *OsSOS1* genes are involved in mediating Na^+^/K^+^ via lowering Na^+^ by defending the cell’s entrance, increasing K^+^ accumulation in rice shoots by transporting Na^+^ from the cytosol to the apoplast [81,82]. *OsHKT1;3*, *OsHAK7*, and *OsSOS1* implicated in conferring salt tolerance exhibit increased levels in the shoots of A91 when exposed to higher salinity (Figure 7b,d,e). Zinc is another component of enzymes involved in the process of photosynthesis, such as theribulose 1,5-bisphosphate carboxylase (RuBPC), that catalyzes the fixation of carbon dioxide under photosynthesis [83]. Additionally, accumulation of higher Ca^2+^ in roots, lower Mg^2+^ and higher Zn^2+^ in shoots also confer the salinity tolerance in A91, while Ca^2+^ in shoots were unchanged compared to WT, Mg^2+^ and Zn^2+^ in roots were reduced (Figure 6), these results suggesting the knockout of *OsbHLH024* significantly improved the tolerance to salinity.

## 4. Materials and Methods

### 4.1. CRISPR/Cas9 Vector Construction and Rice Transformation

The CRISPR-GE website (http://crispr.hzau.edu.cn/CRISPR2, accessed on: 15 January 2018) was used to design the sequences of two targets for *OsbHLH024*. The sequences were launched into double single guide expression cassettes of RNA (sgRNA) by overlapping PCR. Then, the PCR of the first step was performed with U-F and gR-R primers, followed by secondary PCR with appropriate primers for each site, PpS-GGL and PgS-GG2 (for Target 1), Pps-GG2/Pgs-GGR (for Target 2), having a restriction site of BsaI. Ligation of expression cassettes of two sgRNA was made into a vector named pYLCRISPR/Cas9Pubi-H through the Golden Gate system of ligation [51]. Primers of oligonucleotide were used to construct the recombinant vector known as pYLCRISPR/Cas9 (Table S1). The EHA105 strain of *Agrobacterium tumefaciens* was then injected with binary constructs. The rice (Japonica cultivar Nipponbare) was used for the transformation of tissue. The embryogenic calli were transformed using an *Agrobacterium* strain; after 4 weeks of rooting in the media, rice seedlings were shifted to small plastic buckets in the greenhouse, where the controlled day temperature was 30 °C and the night temperature was 26 °C.

### 4.2. Mutation Detection and Assay of Transgene-Free Plant Lines

The CTAB mixture was used to extract the genomic DNA from seedling leaves, and the PCR was carried out with particular primers (Table S1). The characterization of the transgene-free plants was conducted by using plants of the T_1_ generation. SPL-SPR and Cas9 XU-specific primers were used to analyze the genomic DNA via PCR, followed by an agarose gel electrophoresis. The CRISPR/Cas9 vector containing *OsbHLH024* and T_0_ transgenic plants were chosen as positive controls, whereas H_2_O was employed as a negative control. The positive plants with SPL-SPR and Cas9 XU-specific primers were utilized to amplify the expected fragments across Target 1 and 2. The CRISPR/Cas9 positive plant DNA PCR was performed with target primers (F and R). The products of PCR were directly sequenced, using target primers (F and R) with the Sanger method. Positive and negative controls were treated as wild-type (WT) and verified transgenic plants, respectively. In summary, products of PCR were sequenced from the SunYa company.

### 4.3. Phenotypes Observation of the A91 Mutant

WT Nipponbare seeds and CRISPR/Cas9-mediated *OsbHLH024* homozygous mutant (A91) seeds were used. Seeds were first washed 3 times with sterilized water, then surface-sterilized with 75% ethanol for 2 min, shaken with sodium hypochlorite solution (1%) for 20 min, rinsed 5 times in sterile distilled water, and kept for 3 days with tap water, germinated in 37 °C for 1 more day. After germination, seeds were put in the seedbed, and one-month-old plants were shifted to the field. At the maturity stage, we observed the plant phenotypes for statistical analysis.

### 4.4. Total Chlorophyll Content and Fluorescence Determination

The leaf (0.02 g) was sliced into tiny pieces and maintained in a 2 mL tube with 1 mL of acetone overnight at 25 °C. When the leaves were entirely white, samples were centrifuged (10,000 rpm) at 4 °C for 15 min. A Synergy H1 microplate reader was used to detect the absorbance at 663 nm and 645 nm. The supernatant from each sample was collected (200 μL) and used to read absorbance on a 96-plate (BioTek). Calculations were carried out using the formula [84]:

Chlorophyll a = [12.7(A663) − 2.69(A645) × V]/(1000 × W)(1)

Chlorophyll b = [22.9(A645) − 4.68(A663) × V]/(1000 × W)(2)

Chlorophyll (a + b) = Chlorophyll a + Chlorophyll b.(3)

where A = absorbance (wavelengths), V = final volume (chlorophyll extract), W = fresh weight (extracted tissue).

A SPAD 502 plus meter was used to measure the chlorophyll content of fully inflated leaves. The chlorophyll meter, or SPAD meter, is an easy, transportable diagnostic instrument utilized to estimate the leaves’ relative chlorophyll content [85]. The OS-30p (product of OPTI-Sciences, Hudson, NH, USA) was used to calculate the fluorescence of chlorophyll. Dark adaption of plants was confirmed for 30 min prior to quantification at night. The value (*Fv/Fm*) was determined using the prior technique [86].

### 4.5. Salt Stress Experiments

For the salt experiment, seeds were first cleaned 3 times with sterilized water, followed by a surface-sterilization with 75% ethanol for 2 min, then 20 min of shaking with a 1% sodium hypochlorite solution, and finally washed 5 times and kept in a 0.5% H_2_O_2_ solution for 2 days to break dormancy. Seeds were kept in tap water at 37 °C until the occurrence of germination. The germinated seedlings were subjected to a 0.3 g/L Yoshida nutrient solution [87]. At this stage, seedlings were grown at 28 °C for 14 h of light and 10 h of darkness for 3 days. Thereafter, the seedlings were shifted to a 0.5 g/L Yoshida nutrient solution up to 21 days. Treatments with NaCl 150 mM solution started at 21-day-old seedlings for 7 days. After that, we phenotyped the seedlings within 7 days of recovery. Solutions were changed every 3 days.

### 4.6. Antioxidants Measurements

After 7 days of salt treatment (150 mM NaCl), 0.2 g of shoot, and 0.1 g of root samples were harvested in liquid nitrogen. The enzymes were extracted under freezing conditions (liquid nitrogen) and homogenized in a 50 mM (potassium buffer solution) KBS (pH 7.8). Samples were centrifuged for 15 min at 13,000 rpm. The supernatant was collected in 2 mL sterilized tubes at 4 °C. The activities of SOD and POD were spectrophotometrically measured from the extracted supernatant. The activity of POD was measured according to the protocol of Zhou and Leul [88]. The reaction solution of 210 µL included 15 µL distilled water, 5 µL enzyme extract and a 190 µL combination of 1% guaiacol and 0.4% H_2_O_2_ in the presence of 50 mM KBS (pH 7.0). The Synergy H1 microplate (BioTek) reader was used at 470 nm to record variations in absorbance associated with guaiacol oxidation (ε,constant =25.5 mM^−1^ cm^−1^) [89]. For the analysis of SOD, the volume of enzyme necessary to achieve a 50% reduction of the nitroblue tetrazolium (NBT) degradation was used to calculate the SOD activity of one unit. A total of 210 µL reaction solution, 20 µL enzyme extract and 190 µL mixture of NBT 75 μM, Na_2_EDTA 0.1 mM, Methionine 13 mM with riboflavin 2 μM were prepared in 50 mM KBS buffer (pH 7.8). Around 4000 lux light was used to expose samples for 20 min, to achieve the reaction of SOD, and urgently covered with fuel paper to terminate the reaction, as well as blank control in the dark, and immediately measured at 560 nm wavelength by the Synergy H1 microplate reader (BioTek) [90].

### 4.7. Measurements of the Lipid Peroxidation (MDA) and H_2_O_2_

Lipid peroxidation was examined based on malondialdehyde (MDA). To determine H_2_O_2_ content and MDA, 0.2 g shoots and 0.1 g roots were extracted with trichloroacetic acid (0.1% TCA) under cold conditions. A combination of 125 μL enzyme extract and a 500 μL mixture of thiobarbituric acid (0.5%) and trichloroacetic acid (20%) was used to produce the reaction under 95 °C (30 min), and the reaction was stopped by cooling the samples on ice. All samples were centrifuged for 10 min, at 10,000 rpm. The Synergy H1 microplate reader (BioTek) was used to assess the reaction at 532 nm and 600 nm wavelengths (BioTek) [91]. To determine H_2_O_2_ content, 50 µL supernatant was put together with 50 µL of 10 mM KBS (pH 7.0) and 100 µL of 1 M potassium iodide (KI). The H_2_O_2_ was measured at a wavelength of 390 nm. The quantity of H_2_O_2_ in the sample was calculated using a calibration curve [90] (Figure S4). 

### 4.8. Histochemical Staining

The NBT and DAB staining were implemented as previously presented [92] with modifications. To identify superoxide radicals (O_2_^−^), 0.2% NBT was produced with 50 mM KPB (pH 7.5), and to identify H_2_O_2_, staining with DAB (solution 1 mg/1 mL) was used. For staining, both the leaves of WT and the A91 were collected after 4 days of 150 mM stress. The samples were kept in the dark (for DAB 2 days and NBT 1 day). Six leaves from three different plants were chopped into small pieces and placed in a 5 mL tube with a staining reagent for each treatment. After the period described above, the leaves were stored in 100% ethanol for 1 day to eliminate the color due to chlorophyll. Leaves were photographed using a microscope named LEICA MZ 95.

### 4.9. Gene Expression Analysis

For evaluation of the expression pattern of *OsbHLH024*, eight different tissues of shoots (7 days), roots (7 days), shoots (21 days), roots (21 days), leaf sheath, leaf blade, flag leaf angle, and seed (14 days) after pollination were collected for RNA extraction. Tissues of shoots and roots of plants without/with 150 mM NaCl treatments were collected for RNA extraction. The RNA samples were extracted by TRIzol^®^ reagent (Ambion, Carlsbad, CA, USA), and around 1 g (RNA) was utilized to synthesize (cDNA) by the HiScript II-RT Supermix qPCR (+gDNA wiper) kit of the Vazyme company. A Hieff qPCR SYBER green master mix kit was applied to the Roche Light Cycler^®^ 96 (Roche, Basel, Switzerland). The relative expression levels were assessed by applying the double delta system [93], and *OsActin1* was adjusted as an internal control.

### 4.10. Elements Analysis

The contents of Na^+^, K^+^, Ca^2+^, Mg^2+^, and Zn^2+^ were measured with modification as described previously [94]. Samples (roots and shoots) were first dried for two and half days at 70 °C, followed by a digestion using 68% HNO_3_. Three steps were performed, including 100 °C, 120 °C, and 140 °C, each step for 2 h. The 0.05 g of dried sample was digested with 2 mL of HNO_3,_ and the volume was calibrated to 50 mL by adding the distilled water. Finally, elemental analysis results were estimated from a flame photometer (FP-6410).

## 5. Conclusions

In conclusion, this study revealed that the *OsbHLH024* negatively regulates the functions of Na^+^ and K^+^ transporter genes, and the *osbhlh024* mutant (A91) showed a salinity tolerant phenotype. Ion homeostasis (specifically Na^+^ and K^+^) of *osbhlh024* mutant leads to more tolerance, and antioxidants control ROS by suppressing the higher accumulation of MDA and H_2_O_2_. Moreover, the physiological balance of chlorophyll and photosynthesis confers the tolerance level. Therefore, *OsbHLH024* confers a promising tool to improve the tolerance of rice to salt stress, which will bear insights into the *OsbHLH024*-mediated regulatory apparatus of salinity tolerance and provide a better perception of the importance of the *OsbHLH024* gene in relation to abiotic stress.

## Data Availability

The data presented in this study are available within the article.

## Supplementary Materials

The following supporting information can be downloaded at: https://www.mdpi.com/article/10.3390/plants11091184/s1, Figure S1. A linear form of the sgRNA vectors; Figure S2. Mutation process showing chromatograph of *OsbHLH024*; Figure S3: The growth characteristics of the A91 and WT after 7 days of 150 mM NaCl stress of 21-day-old seedlings; Figure S4: Standard curve for H_2_O_2_ determination; Table S1: Primers used in this study; Table S2: The agronomic traits of A91 and the WT in the field.
