# Supplementary material for: CRISPR/Cas9 Mediated Knockout of the OsbHLH024 Transcription Factor Improves Salt Stress Resistance in Rice (Oryza sativa L.)
Source: Plants (Basel). 2022 Apr 27;11(9):1184. doi: 10.3390/plants11091184 (PMC9101608; doi:10.3390/plants11091184)
Supplement: Supplementary file 1 [file plants-11-01184-s001.zip › plants-1675741-supplementary.pdf]

Table S1. Primers used in this study

Primers	Sequence (5´-3´)
**Recombinant pYLCRISPR/Cas9 vector construction**	
U-F	CTCCGTTTTACCTGTGGAATCG
gR-R	CGGAGGAAAATTCCATCCAC
Pps-GGL	TTCAGAGGTCTCTCTCGACTAGTATGGAATCGGCAGCAAAGG
Pgs-GG2	AGCGTGGGTCTCGTCAGGGTCCATCCACTCCAAGCTC
Pps-GG2	TTCAGAGGTCTCTCTGACACTGGAATCGGCAGCAAAGG
Pgs-GGR	AGCGTGGGTCTCGACCGACGCGTATCCATCCACTCCAAGCTC
Target-1OsbHLH024	ATTCCACTCACTCAACCGAA
Target-2 OsbHLH024	GATGTCGGAGAGGAAGAGGA
**Mutation detection**	
OsbHLH024T1F1	CGTGCCGATGGATCAGAAG
OsbHLH024T1R1	GGGATTGATTTCCTCTTAATCAGC
**Transgenic plant identification**	
XUCas9-F1	TGCTCACCCTGTTGTTTGGT
XUCas9-R1	TCATGTGAGCGAGAGCAAGG
SPL	GCGGTGTCATCTATGTTACTAG
SPR	CCGACATAGATGCAATAACTTC
**qRT-PCR**	
OsHKT1;1 F	ATTAGCAGAGCACTGTGGAGGAA
OsHKT1;1 R	CCGACGAACCCGTAGGAAG
OsHKT1;3 F	CAGTTCATCTACCAAAACAATCCA
OsHKT1;3 R	AATACCTCACCACCAATCAGCA
OsHKT1; 5 F	TGCCACCTTACACCACTTTCG
OsHKT1; 5 R	TGCCATACGCACTGATAACCTC
OsHAK7 F	TGCTGTGACACTTGGTTTCC
OsHAK7 R	AAATAACAAGGCGAGCAGGA
OsSOS1F1	GACACTGGGAATGTTCTATGC
OsSOS1R1	CCATGAAGCACCGTGCCTCTCG

OsLEA3 F1	AATGATTTCCCTTTGGGTC
OsLEA3 R1	CATCAGTACACATCACCCA
OsACTIN1 F (35s)	CTCAGCACATTCCAGCAGATGTG
OsACTIN1 R (35s)	GATAACAGCTCCTCTTGGCTTAGC

Table S2. The agronomic traits of A91 and the WT in the field

Traits	WT	A91 Mutant
Plant height (cm)	76.21 ± 0.69	81.02 ± 0.53**
Panicle length (cm)	21.67±0.35	21.61 ±0.27
Primary branches per panicle	8.57 ±0.29	8.87± 0.16
Secondary branches per panicle	21.63±0.49	22.4±0.63
Flag leaf length (cm)	32.49±0.80	33.25±0.68
Panicles per plant	8.67±0.45	8.27±0.49
Grains per panicle	89.83±2.39	93.33±2.13
Seed setting rate (%)	78.06±2.1	78.89±1.3
1000- grain weight (g)	24.42±0.29	24.47±0.37
Grain length (mm)	7.28±0.07	7..37±0.05
Grain width (mm)	3.25±0.02	3.27±0.02
Grain thickness (mm)	2.14±0.01	2.17±0.01
Tiller biomass (g)	7.63±0.40	8.76±0.26*

Values are expressed as means ± SE; n=10 biological replicates; * and ** denote significant *t*-test results at $P < 0.05$ and $P < 0.01$, respectively.

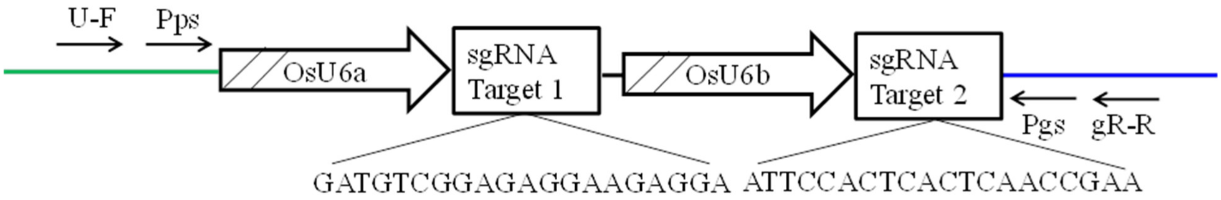

Figure S1. A linear form of the sgRNA vectors. The target-1 and target-2 were driven by OsU6a and OsU6b promoters, respectively by PCRs using primers U-F/gR-R and Pps/Pgs.

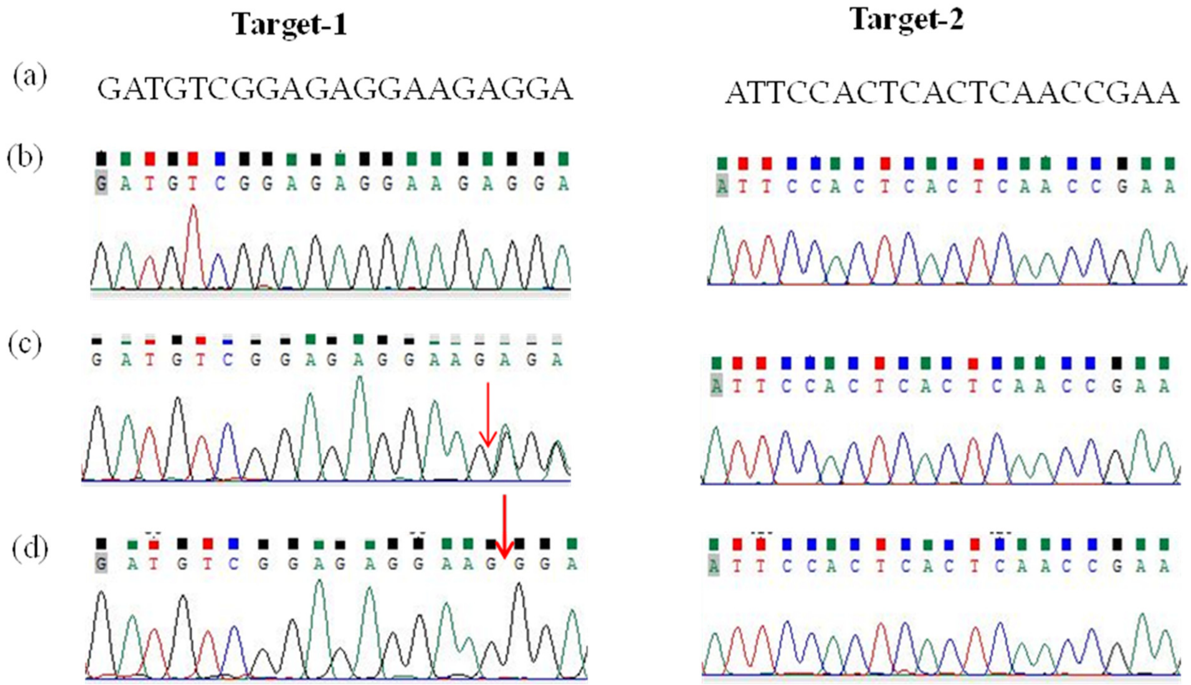

Figure S2. Mutation process showing chromatograph of *osbHLH024*. (**a**) Wild-type sequence of two targets; (**b**) two targets in EHA105 strain of *Agrobacterium tumefaciens*; (**c**) heterozygous mutation in T0 generation, the red arrow indicates heterozygous starting position; (**d**) sequence of homozygous T1 mutant (A91), the red arrow indicates deletion of "A."

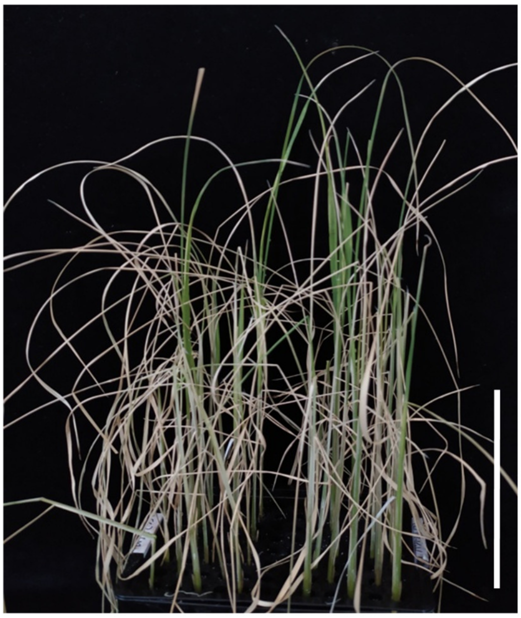

WT **A91**

Figure S3. The growth characteristics of the A91 and WT after 7 days of 150 mM NaCl stress of 21-day-old seedlings. Scale bar 10 cm.

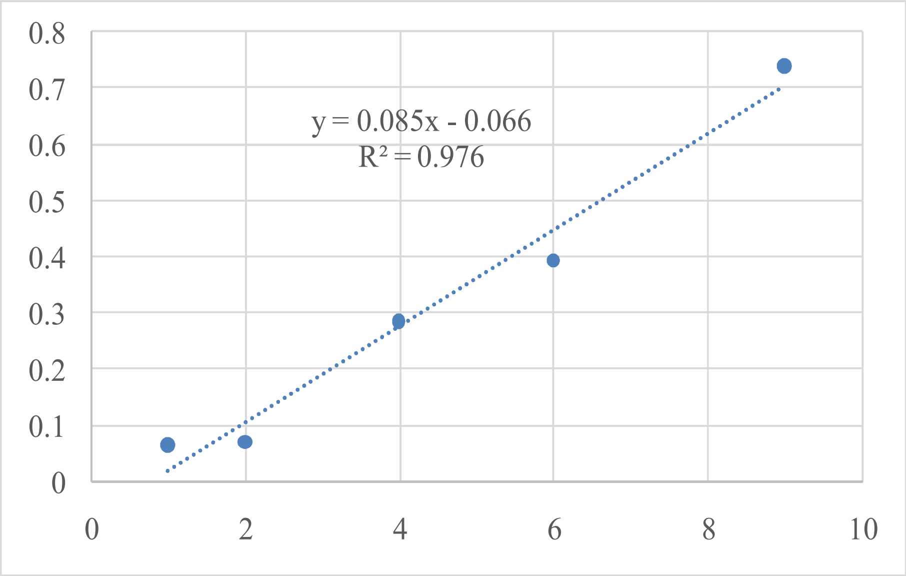

Figure S4. Standard curve for H_2O_2 determination.
